# The Clinical Features and Outcomes of Pseudocirrhosis in Breast Cancer

**DOI:** 10.3390/cancers16162822

**Published:** 2024-08-12

**Authors:** Edward Phillips, Mantegh Sethi, Surammiya Vasanthakumar, Gina Sherpa, Stephen Johnston, Marina Parton, Emma Kipps, Nicholas C. Turner, Matthew Foxton, Alicia Okines

**Affiliations:** 1 Breast Unit, The Royal Marsden Hospital, London SW3 6JJ, UK; edward.phillips@nhs.net (E.P.); suramvk@yahoo.co.uk (S.V.); gina.sherpa@nhs.net (G.S.);; 2 Department of Surgery, University Hospitals Birmingham, Birmingham B15 2GW, UK; sethim@doctors.org.uk; 3 Department of Gastroenterology, Chelsea and Westminster Hospital, London SW10 9NH, UK

**Keywords:** liver, metastases, cirrhosis, HER2, pseudocirrhosis

## Abstract

**Simple Summary:**

Pseudocirrhosis is a nodularity in the liver that it is typically associated with breast cancer liver metastases, and may occur as a response to chemotherapy and other systemic anticancer treatments. The types of patients who develop pseudocirrhosis, the treatments that they have received, and their outcomes are not well known. This study reviewed 170 patients with a diagnosis of pseudocirrhosis. A variety of different anticancer treatments were received, with taxanes (74.7%) and capecitabine (67.1%) being the most common. The median time between diagnosis of liver metastases and diagnosis of pseudocirrhosis was 17.1 months. The median overall survival once diagnosed with pseudocirrhosis was 7.6 months and patients with HER2+ disease had a statistically significant longer overall survival. To our knowledge, this is the largest dataset of pseudocirrhotic patients that has been published. It provides information to patients and clinicians on risk factors to develop pseudocirrhosis, and prognosis in different subtypes.

**Abstract:**

Pseudocirrhosis is a diffuse nodularity of the liver that radiologically mimics cirrhosis but is a distinct pathological process. It is seen almost exclusively in patients with liver metastases and may represent a response to systemic treatment. Data on the risk factors for pseudocirrhosis and outcomes are limited. In total, 170 patients with a diagnosis of breast cancer and pseudocirrhosis in a 10-year period were identified and retrospectively analysed. Data were collected on baseline patient characteristics, treatments received, and outcomes. Median time between diagnosis of liver metastases and diagnosis of pseudocirrhosis was 17.1 months (range, 0–149 months). In total, 89.4% of patients received chemotherapy between their diagnosis of breast cancer liver metastases and their diagnosis of pseudocirrhosis, most commonly a taxane (74.7%) or capecitabine (67.1%), and the median treatment lines received was 3. Median OS from first diagnosis of pseudocirrhosis was 7.6 months (95% CI: 6.1–9.6 months) and was longer in patients with HER2+ disease at 16.7 months (95% CI: 6.4–32.9 months), which was statistically significant. In our study, pseudocirrhosis occurred in the presence of liver metastases and was associated with a poor prognosis. HER2+ patients with pseudocirrhosis had a better prognosis than other subtypes, but we did not identify other significant predictors of survival. Chemotherapy was not a prerequisite for pseudocirrhosis development, although the majority of patients had received at least one line of chemotherapy before pseudocirrhosis was diagnosed.

## 1. Introduction

Pseudocirrhosis is defined as the diffuse nodularity of the liver seen in patients with known liver metastases, but without one of the underlying causes of chronic liver disease seen in true cirrhosis [1]. The features of pseudocirrhosis were first described as hepar lobatum, due to the healing granulomas and scar contraction seen in tertiary syphilis [2]. Later, pseudocirrhosis was linked to breast cancer with the involvement of liver metastases (hepar lobatum carcinomatosum) [3]. Clinical features of pseudocirrhosis mimic those of cirrhosis due to chronic liver disease including portal hypertension, ascites, oesophageal varices, hepatic encephalopathy, and splenomegaly [4]. The reported prevalence of pseudocirrhosis with evidence of portal hypertension varies significantly in the literature, ranging from 38% [5] to 81% [6] of patients with breast cancer liver metastases.

The histopathological processes seen in pseudocirrhosis are distinct from true cirrhosis. True cirrhosis is characterised by fibrotic septa linking portal tracts together, often called bridging fibrosis [7], which is not seen in pseudocirrhosis [8]. Histopathological features of pseudocirrhosis are less well understood. Desmoplastic reactions have been observed, where fibrous tissue develops adjacent to areas of sinusoidal breast cancer infiltration [9]. Another histopathological feature that has been associated is nodular regenerative hyperplasia, where regenerative nodules form which can compress the liver parenchyma and cause sinusoidal congestion [10]. It has been suggested that it could be a result of chemotherapy-induced liver injury [11] or as a response of hepatic tumours to treatment leading to fibrosis and nodular regeneration [4].

Pseudocirrhosis most commonly develops following systemic chemotherapy for breast cancer liver metastases (BCLM). However, pseudocirrhosis has also been described in chemotherapy-naïve patients who have only received endocrine therapy for metastatic breast cancer [12]. Although breast cancer is the most common malignancy leading to pseudocirrhosis, it has also been linked to the treatment of liver metastases in other cancers, including oesophageal cancer [13], colorectal cancer [14], medullary thyroid cancer [15], and pancreatic cancer [16].

The receptor status of a breast cancer is important in determining treatments and prognosis, and may have a role in the development and outcomes of pseudocirrhosis. The likelihood of tumour shrinkage also varies by receptor type [17]. Oestrogen and progesterone receptors are the most commonly expressed and these breast cancers are considered hormone receptor positive (HR+) breast cancers. Typically, they have the best prognosis [18], but conversely, are less likely to have a response to first line chemotherapy [17]. If the human epidermal growth factor 2 (HER2) receptor is expressed, then patients can receive HER2-targeted therapies with chemotherapy, with excellent response rates expected and prolonged median survival times reported [19]. HER2-positive cancers can be further subdivided into either HER2+/HR+ (triple positive) or HER2+/HR− cancers. Finally, triple negative cancers express neither HER2 nor hormone receptors. These cancers typically have the worst prognosis but often have good, although frequently short-lived, responses to chemotherapy [17].

Previous case series of pseudocirrhosis in advanced breast cancer [5,20,21] have reported limited information on the treatment regimens that preceded development of pseudocirrhosis. Our study of a single centre experience of pseudocirrhosis in advanced breast cancer was designed to evaluate the patient characteristics, previous anticancer therapies, rate of complications, and outcomes of patients with pseudocirrhosis.

## 2. Materials and Methods

### 2.1. Patients

Electronic medical records were searched for eligible patients who had been treated for breast cancer at the Royal Marden Hospital, a large tertiary cancer centre in the UK. All patients with a diagnosis of breast cancer between November 2008 and November 2018, who had one of the following terms on a radiology report were assessed for eligibility: pseudocirrhosis, pseudocirrhotic, cirrhosis, cirrhotic, ascites, and portal hypertension. Imaging modalities included were CT, PET, MRI, and ultrasound. Any scan where a diagnosis of pseudocirrhosis was made were included and all scans used the standard imaging protocol for the specific scan type at our hospital. In cases where there was uncertainty on the initial scan or a suboptimal scan had taken place, a confirmatory follow-up scan was done, such as a liver MRI. All imaging had been reported by a specialist oncological radiologist and the report and case notes were assessed by the medical team for inclusion in the study.

The endpoints of this study comprised time to development of pseudocirrhosis from diagnosis of BCLM, overall survival (OS) from pseudocirrhosis diagnosis, clinicopathological features of patients developing pseudocirrhosis, the number and type of preceding treatment regimens, the time from chemotherapy to pseudocirrhosis, and the frequency of complications of pseudocirrhosis.

### 2.2. Statistical Analysis

Descriptive analyses were undertaken for patient and treatment characteristics, including frequencies, percentages, median, and range. BCLM was defined as patients with a diagnosis of breast cancer and radiologically identified liver metastases. Similarly, pseudocirrhosis was defined as the presence of diffuse nodular changes on the liver identified by CT, MRI, or ultrasound, and reported by a specialist radiologist.

Overall survival time from BCLM to pseudocirrhosis, and time from chemotherapy to pseudocirrhosis, were estimated using Kaplan-Meier methods. The survival endpoints of interest were overall survival from diagnosis of BCLM and OS from diagnosis of pseudocirrhosis, with receptor status log-rank analysis also undertaken. Patients who were lost to follow-up or did not meet the event of interest at last follow-up were right censored. To model the impact of covariates on survival, Cox Proportional Hazards regression was undertaken for the multiple predictor variables collected. Hazard ratios (HR) with 95% confidence intervals (CI) were reported for each covariate. A nominal *p*-value of <0.05 was considered statistically significant, based on a two-tailed hypothesis. All statistical analyses were performed using Python 3.8.2, with packages Numpy, Pandas, and Lifelines [22].

Log-rank tests were undertaken to compare the survival distributions based on receptor status. The number of events required to adequately power the log-rank test were calculated according to Schoenfeld’s formula.

## 3. Results

**Figure 1:**
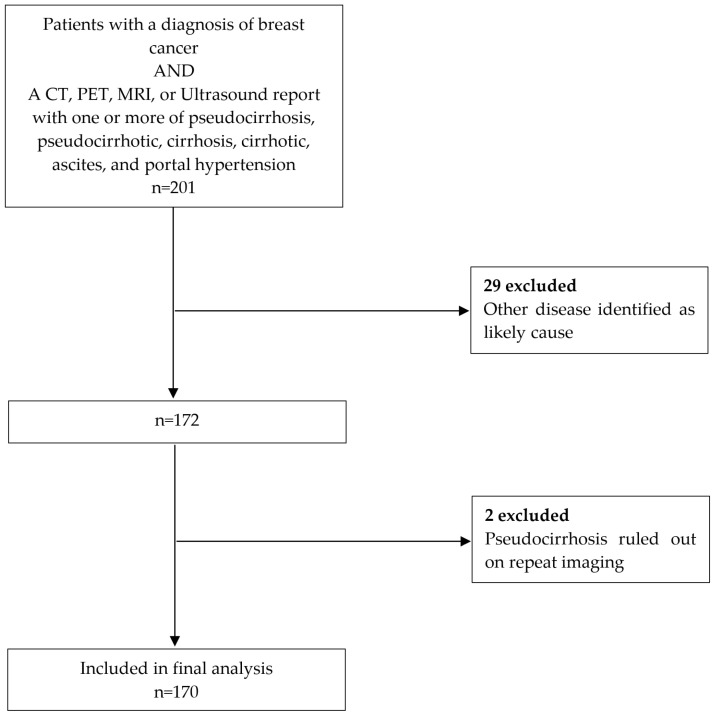
CONSORT flow diagram detailing selection of patients for analysis.

As displayed in Figure 1, all patients who had a diagnosis of breast cancer but had another cause of cirrhosis or pseudocirrhosis were excluded. These patients either had a second malignancy or had chronic liver disease that was incidental to their breast cancer diagnosis. In addition, two patients had one of the aforementioned search terms included on a report, but were deemed not to have pseudocirrhosis on repeat imaging.

### 3.1. Patient Characteristics

In total, 170 eligible patients were identified (Figure 1) over the 10-year period studied. Clinicopathological features are summarised in Table 1. All patients included in the analysis were female, mean age at diagnosis 56.4 ± 12.5 years (range 29.2 to 88.0 years). The majority (84%) of patients were white; 91.2% had invasive ductal carcinoma. The most common imaging modality that was used to first diagnose pseudocirrhosis was CT in 160 patients (94.1%), followed by MRI in 6 (3.5%), and ultrasound in 4 (2.4%). Patient characteristics are summarised in Table 1.

Median time between diagnosis of liver metastases and diagnosis of pseudocirrhosis was 17.1 months (range 0–149 months). Across receptor subtypes, this was longest in HR+/HER2+ patients at 23.5 months, and shortest in triple negative and HR−/HER2+ cancers at 8.1 and 8.9 months, respectively. The most frequent extra-hepatic disease site was skeletal metastases, seen in 81.2% of patients.

### 3.2. Treatment Characteristics

The median number lines of systemic anti-cancer therapy (SACT) received between diagnosis of liver metastases and pseudocirrhosis was 3 (range 0–11, mean of 3.3). The median number of lines of chemotherapy received between diagnosis of liver metastases and pseudocirrhosis was 2 (range 0–7, mean of 2.0). Across receptor subtypes the median number of lines of treatment was 3 in HR+/HER2− cancers (mean 3.5), also 3 in the HR+/HER2+ group (mean 3.2), 2 in the triple negative group (mean 2.3), and 1 in the HR−/HER2+ group (mean 1.7). Table 2 summarises these data as well as the number of lines of chemotherapy.

Not unexpectedly for a cohort of patients with mostly ER-positive disease, the most common treatment was an aromatase inhibitor, received by 51.2% of patients after BCLM diagnosis. In total, 89.4% of patients received chemotherapy, most commonly a taxane (74.7%) or capecitabine (67.1%). All 31 patients with HER2 positive (HER2+) breast cancer received HER2 targeted therapy, and none of the 12 patients with triple negative breast cancer received immunotherapy due to the era in which the patients were treated. Systemic treatments received between the diagnosis of breast cancer liver metastases and diagnosis of pseudocirrhosis, as well as systemic treatments received at time of pseudocirrhosis diagnosis, are summarised in Table 3.

In patients with BCLM, the median time between starting SACT and developing pseudocirrhosis was 16.6 months. The median time between starting chemotherapy and developing pseudocirrhosis was 15.8 months. At the time of pseudocirrhosis diagnosis, 14.6% of patients were receiving endocrine therapy (with a targeted agent in 4.1%) and 70.3% chemotherapy, reflecting that pseudocirrhosis is normally a late complication so would be expected to most commonly occur in patients whose cancers have already developed endocrine resistance.

For seven patients, the diagnosis of BCLM and pseudocirrhosis were synchronous and noted on the same scan. All patients with pseudocirrhosis had received SACT, either chemotherapy or endocrine therapy.

### 3.3. Outcomes

**Figure 2:**
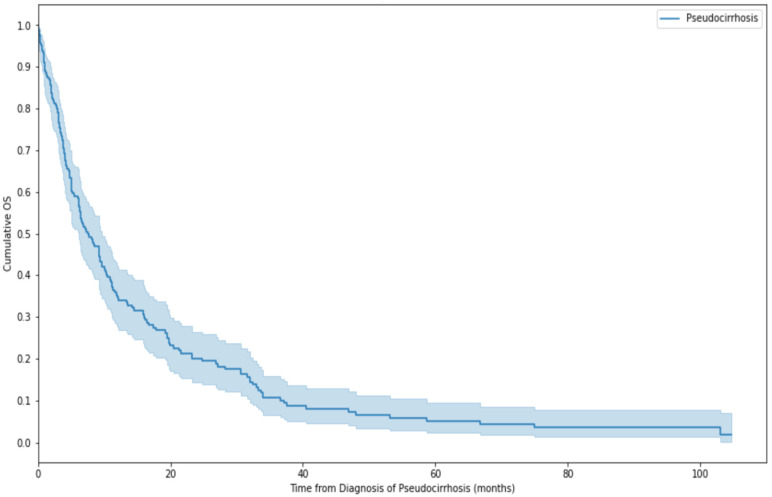
Kaplan-Meier curve demonstrating survival after diagnosis of pseudocirrhosis. Median OS 7.6 months (95% CI: 6.1–9.6 months).

**Figure 3:**
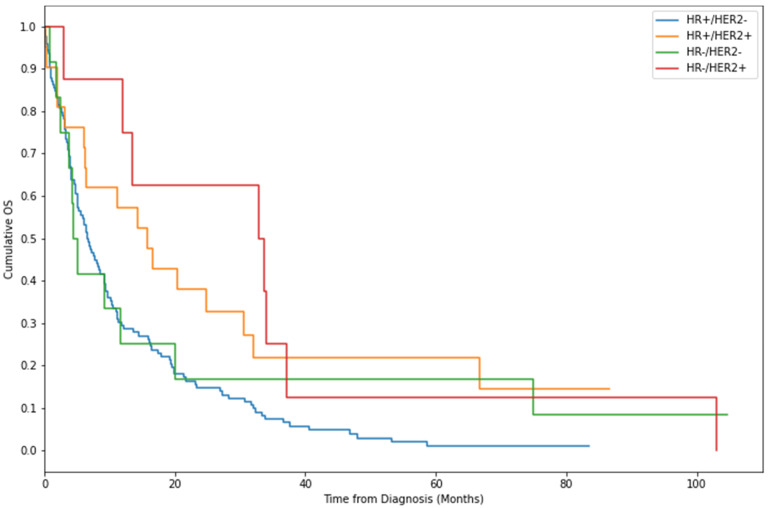
Kaplan-Meier curves demonstrating survival after diagnosis of pseudocirrhosis by receptor subtype. Median OS for each receptor subtype was 6.6 months (95% CI: 5.1–9.2 months) for HR+/HER2−, 15.8 months (95% CI: 6.1–30.6 months) for HR+/HER2+, 33.6 months (95% CI: 3.00–37.2 months) for HR−/HER2+, and 4.4 months (95% CI: 1.8–20.0 months) for HR−/HER2−.

In patients that would later develop pseudocirrhosis, median OS from first diagnosis of BCLM was 27.8 months (95% CI: 24.9–35.5 months). Median OS from first diagnosis of pseudocirrhosis was 7.6 months (95% CI: 6.1–9.6 months). A Kaplan-Meier curve demonstrating survival after diagnosis of pseudocirrhosis is seen in Figure 2. The initially steep gradient of the curve demonstrates that, for a proportion of patients, diagnosis of pseudocirrhosis is a peri-terminal event. However, many patients survived for several years with the diagnosis of pseudocirrhosis, at least in part due to the broad variation in prognosis between the different subtypes of breast cancer. Median OS was longer in patients with HER2+ disease at 16.7 months (95% CI: 6.4–32.9 months) and when further categorised into only HR+/HER2+ disease was 33.6 months (95% CI: 3.00–37.17 months). It was shortest in patients with triple negative cancers at 4.4 months (95% CI: 1.80–19.97 months). On log-rank testing, HER2+ patients had significantly better outcomes compared to patients with triple negative or ER+/HER2− disease, (*p* < 0.05). Although numerically different, there was no statistically significant difference between the survival of patients with triple negative and those with ER+/HER2− disease (*p* = 0.71). Kaplan-Meier curves for each subtype are shown in Figure 3.

### 3.4. Complications of Pseudocirrhosis

**Figure 4:**
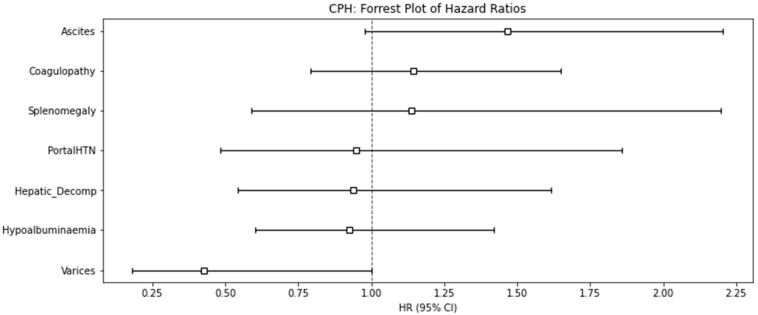
Forest plot of hazard ratios for death with 95% confidence intervals for features of hepatic dysfunction.

Table 4 details the features of liver dysfunction seen in pseudocirrhosis. The most common complication of pseudocirrhosis was hypoalbuminaemia, noted in 54.7% of patients. Albumin levels from the last 4 weeks of a patient’s life had been excluded to reduce the contribution of other causes for hypoalbuminemia, such as malnutrition. Ascites occurred in 50.6%, requiring intervention with ascitic drainage in the majority of patients. Radiological features of portal hypertension, including ascites and splenomegaly, were reported in 59.4%. However, only 11.8% of patients were deemed to have hepatic decompensation, which was defined for this study by loss of synthetic function, hyperbilirubinaemia, or hepatic encephalopathy. No variceal bleeds were recorded. On multivariate analysis, no single feature of hepatic dysfunction was associated with survival. Figure 4 shows a forest plot for the hazard ratios of death for each of the features of hepatic dysfunction.

## 4. Discussion

A number of different mechanisms for the development of pseudocirrhosis have been postulated. One theory is that it is a desmoplastic reaction of the liver to SACT in the presence of metastases [3,23]. Desmoplasia describes the development of fibrotic tissue and can be seen as a reaction to cell death, in this case in response to chemotherapy. This is supported by liver biopsies taken after treatment with chemotherapy, which have shown desmoplastic reactions [24]. The presence of liver metastases would be, therefore, required for pseudocirrhosis to develop. This is also supported by a study of the CT scans of patients with pseudocirrhosis, which found that the pseudocirrhotic changes were only seen in parts of the liver with BCLM [25]. Correspondingly, and consistent with other studies [12], we found no patients who developed pseudocirrhosis in the absence of BCLM.

Another theory is that chemotherapy induces a state of nodular regressive hyperplasia in patients either with or without BCLM, leading to contraction of the liver capsule and the pseudocirrhotic appearance on imaging [25]. However, in our study, 10% of patients had never received chemotherapy for metastatic disease but developed a pseudocirrhotic appearance. These patients had, however, all received a systemic therapy for breast cancer, most commonly endocrine therapy with or without a CDK4/6 inhibitor. It may be that any systemic treatment of BCLM can induce development of fibrosis and that the type of SACT, and the resultant mechanism of cell death, is less important. However, a single case report of a patient presenting with pseudocirrhosis and liver metastases 14 years after initial breast cancer treatment suggests that the condition can also rarely occur even in the absence of systemic therapy [11], or perhaps was present following initial treatment, but never clinically apparent. Similarly, three patients in our study did not receive any SACT between their diagnosis of BCLM and pseudocirrhosis. One patient had never received any SACT for metastatic breast cancer, but had received adjuvant chemotherapy previously.

Another question is whether pseudocirrhosis can happen in the absence of BCLM. Chemotherapy-induced liver injury has been shown to cause nodular regenerative hyperplasia in the absence of metastases [26]. However, pseudocirrhosis is infrequently seen in non-breast cancers despite extensive use of chemotherapy [27]. Our data found no cases of pseudocirrhosis without liver metastases, and therefore, pseudocirrhosis in the absence of liver metastases is either not possible or sufficiently rare that we did not detect it in our patient population due to the sample size. Consistent with previous datasets, patients with triple negative and other grade 3 tumours were infrequent in our cohort of patients with pseudocirrhosis [21,28]. This is likely to reflect their more aggressive disease course and poorer prognosis and therefore insufficient time to develop this complication, rather than a true lack of association.

Our data found that HR+/HER2+ cancers may take longer to develop pseudocirrhosis than other receptor subtypes, although there are small numbers of patients in some of these groups. It has been speculated that the shrinking of BCLMs are associated with pseudocirrhosis development [4]. Therefore, breast cancer subtypes that are most responsive to first line treatment may be more likely to develop pseudocirrhosis. In the neoadjuvant setting, HR−/HER2+ have the highest response rates, followed by triple negative cancers, then HR+/HER2+, and finally HR+/HER2− [29]. We suggest that the shorter time to develop pseudocirrhosis is linked to these higher response rates.

We have confirmed a previous report that patients with HER2+ BCLM and pseudocirrhosis had a significantly better OS than those with HER2-negative disease [30]. Our data also found that triple negative cancers have a shorter median OS of 4.4 months. This is most likely explained by the varying prognosis of the different receptor subtypes, with HER2+ cancers having the best prognosis and triple negative cancers the worst [31]. Our data support previous studies that the presence of pseudocirrhosis on imaging is a poor prognostic sign. Median OS from diagnosis of pseudocirrhosis was 7.6 months (95% CI: 6.1–9.6 months) in our series, similar to the median OS of 7.9 months reported in another single centre experience of 120 patients [6], previously the largest dataset published. Other smaller studies found a median OS from date of pseudocirrhosis diagnosis of 10 months in 86 patients [21] and 8.5 months in 48 patients, both single-centre studies [28], to as low at 2.3 months from date of first liver metastasis [12].

Although pseudocirrhosis is a distinct pathological pattern to cirrhosis, it shares many of the same clinical complications. Hypoalbuminaemia and ascites were common, being seen in 54.7% and 50.6%, respectively, in our study. However, we found a lower proportion of patients with other features of liver disease, such as oesophageal varices, than has been reported in other studies [6,21]. This likely represents different practices and varying thresholds for investigations such as gastroscopy, which is rarely undertaken in our practice in the absence of upper GI bleeding. Previous studies have reported the presence of ascites as a poor prognostic factor in pseudocirrhosis [6], which we were unable to confirm. Previously, a significantly shorter prognosis has been reported in patients with varices, due to pseudocirrhosis in a series of 106 patients of whom 32 had varices diagnosed [30]. Unexpectedly, (*p* = 0.05), varices appeared to be a protective feature in our study, with patients having a numerically, although not statistically significant, longer overall survival. This was likely confounded by the selection of patients with a good prognosis for gastroscopy, meaning a significant proportion of patients with varices remained undiagnosed.

To our knowledge, this is the largest series of breast cancer patients with pseudocirrhosis that has been reported to date. However, our study has a number of limitations, including the retrospective collection of data. Furthermore, we did not include a control group in our study design, and therefore, we cannot determine any predisposing factors for pseudocirrhosis development. Additionally, eligibility for our study did not require histological confirmation of pseudocirrhosis diagnosis, nor testing for hepatitis B and C viral serology, haemochromatosis, or auto-immune causes of cirrhosis. Therefore, although we excluded patients with known chronic liver disease, some patients included may have had true cirrhosis from undiagnosed causes. Another important limitation of our study is that only 9.7% of HR positive HER2 negative patients received a CDK4/6 inhibitor during the studied period. This is likely because the data collection was between November 2008 and November 2018, and therefore, the majority of patients received treatment before the approval of CDK4/6 inhibitors in the UK in November 2016. Additionally, the majority of patients received one or more lines of treatment prior to developing BCLM, so some may have received a CDK 4/6 inhibitor as first- or second-line treatment, prior to developing BCLM. These are data which we did not collect in our study.

Future studies may answer how more modern SACTs, such as CDK4/6 inhibitors, antibody drug conjugates, and immunotherapy, may be associated with pseudocirrhosis. There is also a lack of understanding of the histopathological changes that the liver undergoes during pseudocirrhosis development. A study which compared liver biopsies before and after pseudocirrhosis diagnosis across different SACTs may answer some of these questions.

## 5. Conclusions

To our knowledge, this is the largest study of patients with pseudocirrhosis. In the timeframe between diagnosis of first liver metastasis and diagnosis of pseudocirrhosis, 10.6% of patients received no chemotherapy and several patients never received chemotherapy in the metastatic setting. Therefore, pseudocirrhosis is not solely caused by a response to chemotherapy. Triple negative and Grade 3 cancers were less likely to be diagnosed with pseudocirrhosis, but this may be due to their rapid clinical course.

Our data found an OS of 7.6 months (95% CI: 6.1–9.6 months), which is similar to previously reported figures [18,20]. We identified a longer median OS of patients with HER2+ disease at 16.7 months (95% CI: 6.4–32.9 months), which was statistically significant. This has not been reported in previous datasets. These findings suggest that avoiding chemotherapy is not sufficient to avoid pseudocirrhosis. Pseudocirrhosis is a cause of many types of liver dysfunction. Clinicians and patients should be aware of these and monitor for signs and symptoms accordingly.

## Figures and Tables

**Table 1:** Patient characteristics.

Feature	*n* (%)
**Ethnicity**	
White	143 (84)
Asian	10 (6)
Black	3 (2)
Chinese	3 (2)
Unknown	11 (6)
**Histological subtype**	
Invasive ductal carcinoma	155 (91.2)
Invasive lobular carcinoma	14 (8.2)
Mucinous	1 (0.6)
**Histological grade at diagnosis**	
Grade 1	4 (2.4)
Grade 2	88 (51.8)
Grade 3	67 (39.4)
Unknown	11 (6.5)
**Receptor Status**	
Hormone receptor positive	148 (87.1)
HER2 positive	31 (21.8)
Triple negative	12 (7.0)
**Extrahepatic sites of metastatic disease**	
Skeletal	138 (81.2)
Lung	48 (28.2)
Nodal	36 (21)
Brain	25 (14.7)
**Imaging modality used for pseudocirrhosis diagnosis**	
CT	160 (94.1)
MRI	6 (3.5)
Ultrasound	4 (2.5)

**Table 2:** Lines of SACT, lines of chemotherapy and time to develop pseudocirrhosis by receptor subtype.

Receptor Status	Lines of SACT		Lines of Chemotherapy		Median Time from First BCLM to Pseudocirrhosis (Months)
	Median	Mean	Median	Mean	
All patients (*n* = 170)	3	3.3	2	2.0	17.1
HR+/HER2− (*n* = 127)	3	3.5	2	2.2	17.7
HR+/HER2+ (*n* = 21)	3	3.2	1	1.8	23.5
HR−/HER2+ (*n* = 10)	1	1.7	1	1.4	8.9
Triple negative (*n* = 12)	2	2.3	2	1.7	8.1

**Table 3:** Systemic treatments received between development of liver metastases and pseudocirrhosis.

Regime	Number of Patients Receiving Treatment at Any Point between Diagnosis of BCLM and Pseudocirrhosis *n* (%)	Number of Patients Receiving Treatment at Time of Pseudocirrhosis Diagnosis *n* (%)
**Aromatase inhibitor**	87 (51.2)	17 (10.0)
Letrozole	43 (25.3)	8 (4.7)
Anastrozole	5 (2.9)	2 (1.2)
Exemestane	45 (26.5)	3 (1.7)
Tamoxifen	38 (22.4)	4 (2.4)
Fulvestrant	33 (19.4)	9 (5.3)
**CDK 4/6 inhibitor**	12 (7.1)	7 (4.1)
Palbociclib	8 (4.7)	6 (3.5)
Ribociclib	3 (1.8)	0 (0.0)
Abemaciclib	1 (0.6)	1 (0.6)
**Anti-HER2 therapy**	31 (18.2)	20 (11.8)
Trastuzumab	30 (17.6)	10 (5.9)
Pertuzumab	7 (4.1)	3 (1.7)
Trastuzumab emtansine	7 (4.1)	6 (3.5)
Lapatinib	11 (6.5)	1 (0.5)
Neratinib	2 (1.2)	0 (0.0)
**Chemotherapy**	152 (89.4)	135 (79.4)
Capecitabine	114 (67.1)	37 (21.8)
Epirubicin	25 (14.7)	10 (5.9)
Taxane	127 (74.7)	40 (23.5)
Cyclophosphamide	16 (9.4)	3 (1.7)
Eribulin	36 (21.2)	18 (10.6)
Platinum	37 (21.8)	7 (4.1)
Gemcitabine	13 (7.6)	3 (1.7)

**Table 4:** Features of liver dysfunction seen in pseudocirrhosis.

Feature	*n* (%)
Albumin < LLN	93 (54.7)
Ascites	86 (50.6)
Portal hypertension	25 (14.7)
Spelnomegaly	11 (6.5)
Oesophageal varices	7 (4.1)
Hepatic decompensation	20 (11.8)

## Data Availability

The data presented in this study are available on request from the corresponding author.
